# easyPheno: An easy-to-use and easy-to-extend Python framework for phenotype prediction using Bayesian optimization

**DOI:** 10.1093/bioadv/vbad035

**Published:** 2023-03-22

**Authors:** Florian Haselbeck, Maura John, Dominik G Grimm

**Affiliations:** Technical University of Munich, Campus Straubing for Biotechnology and Sustainability, Bioinformatics, Straubing 94315, Germany; Weihenstephan-Triesdorf University of Applied Sciences, Bioinformatics, Straubing 94315, Germany; Technical University of Munich, Campus Straubing for Biotechnology and Sustainability, Bioinformatics, Straubing 94315, Germany; Weihenstephan-Triesdorf University of Applied Sciences, Bioinformatics, Straubing 94315, Germany; Technical University of Munich, Campus Straubing for Biotechnology and Sustainability, Bioinformatics, Straubing 94315, Germany; Weihenstephan-Triesdorf University of Applied Sciences, Bioinformatics, Straubing 94315, Germany; Technical University of Munich, Department of Informatics, Garching 85748, Germany

## Abstract

**Summary:**

Predicting complex traits from genotypic information is a major challenge in various biological domains. With easyPheno, we present a comprehensive Python framework enabling the rigorous training, comparison and analysis of phenotype predictions for a variety of different models, ranging from common genomic selection approaches over classical machine learning and modern deep learning-based techniques. Our framework is easy-to-use, also for non-programming-experts, and includes an automatic hyperparameter search using state-of-the-art Bayesian optimization. Moreover, easyPheno provides various benefits for bioinformaticians developing new prediction models. easyPheno enables to quickly integrate novel models and functionalities in a reliable framework and to benchmark against various integrated prediction models in a comparable setup. In addition, the framework allows the assessment of newly developed prediction models under pre-defined settings using simulated data. We provide a detailed documentation with various hands-on tutorials and videos explaining the usage of easyPheno to novice users.

**Availability and implementation:**

easyPheno is publicly available at https://github.com/grimmlab/easyPheno and can be easily installed as Python package via https://pypi.org/project/easypheno/ or using Docker. A comprehensive documentation including various tutorials complemented with videos can be found at https://easypheno.readthedocs.io/.

**Supplementary information:**

Supplementary data are available at *Bioinformatics Advances* online.

## 1 Introduction

Predicting complex traits from genotypic information remains a major challenge in modern biology, whether to reduce costs and accelerate the breeding process in plants and animals or to assess the risk of diseases in humans. So far, existing studies on phenotype prediction in plants (John *et al.*, 2022), animals (Abdollahi-Arpanahi *et al.*, 2020) and humans (Bellot *et al.*, 2018) fail to determine an overall pre-dominant prediction method. Their results show that the prediction performance is highly dependent on the species-trait combination, thus requiring the re-evaluation of various prediction models. Existing phenotype prediction packages, such as BWGS (Charmet *et al.*, 2020), learnMET (Westhues *et al.*, 2022) and G2PDeep (Zeng *et al.*, 2021), are focused on certain pre-processing and prediction methods without straightforward enhancement options or apply a basic hyperparameter search. Despite Python being one of the most popular programming languages, there is currently no comprehensive phenotype prediction package available. We present easyPheno, an easy-to-use and open-source Python framework that enables the rigorous training, comparison and analysis of a variety of phenotype prediction models. More importantly, besides an extensive data pre-processing module, easyPheno leverages Bayesian optimization for an advanced and automatic hyperparameter search. We further designed easyPheno to be user-friendly, also for non-programming-experts, and simplified the integration and benchmarking of new prediction models. As a further plus, easyPheno integrates the simulation of phenoypic data, i.a., to assess newly developed approaches under pre-defined settings. Beyond that, we provide a comprehensive online documentation, including various hands-on tutorials and videos.

## 2 Use cases

easyPheno offers a variety of use cases for end users that might not have programming expertise, e.g. plant or animal breeders, as well as bioinformaticians that aim to develop new prediction models and functionalities. In the following, we outline the main benefits for both user groups.

### 2.1 State-of-the-art and easy-to-use phenotype prediction

**Fig. 1.**
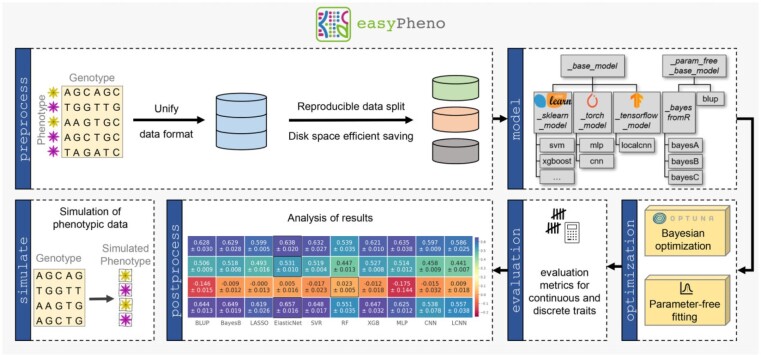
Overview of easyPheno’s main modules. In the preprocess module, various methods are offered to unify, filter and split data to enable a reproducible data pre-processing. Several prediction models from which the user can choose are already integrated. In addition, the design of the model subpackage allows a straightforward extension. Further, parameter-free fitting and state-of-the-art Bayesian optimization for automatic hyperparameter search are included (optimization). To analyze the results, various evaluation metrics for continuous and discrete phenotypes are included, as well as methods for an easy analysis of the prediction results (postprocess). For end users, we offer an easy-to-use command line interface, allowing to run state-of-the-art phenotype prediction with a single-line command. easyPheno is further available as a Python package. Besides a quick integration of new prediction models in a reliable framework, this allows bioinformaticians to benchmark newly developed approaches in a comparable setup. In addition, the simulate module allows to generate synthetic phenotype data, which can be used for a model assessment under pre-defined conditions

easyPheno provides a command line interface only requiring a single-line command to run state-of-the-art prediction approaches on discrete and continuous traits. In Figure 1, we summarize easyPheno’s main packages and visualize the phenotype prediction pipeline. To start the main workflow, users only need to supply a fully imputed genotype matrix and the corresponding phenotype values for one or more traits of interest. easyPheno completely automates the data pre-processing as well as the hyperparameter tuning using state-of-the-art Bayesian optimization techniques, see Section 3 for further details. easyPheno ensures reproducibility of the whole pipeline, e.g. regarding the data split, to allow comparability of results. With its easy-to-use command line interface, easyPheno allows to quickly run comparative studies employing various prediction models ranging from classical genomic selection approaches over machine learning (ML) methods to modern deep learning (DL) architectures. Users can start the pipeline both for multiple prediction models and phenotypes simultaneously. Hence, easyPheno is a powerful tool also for users without ML and programming expertise, enabling them to conduct state-of-the-art phenotype prediction. An exemplary workflow of easyPheno with real and synthetic data is shown in Supplementary Material.

### 2.2 Quick integration and benchmarking of new predictors

Besides a command line interface enabling a straightforward application of state-of-the-art phenotype prediction approaches, easyPheno is offered as a Python package, i.a., to simplify the implementation of new functionalities for developers. Our framework is designed to enable a quick integration of new prediction models, especially for methods based on the most widely applied ML frameworks scikit-learn, PyTorch and TensorFlow. This easy-to-extend design allows bioinformaticians to focus on the model design while relying on thoroughly tested data pre-processing and state-of-the-art hyperparameter optimization. This is particularly interesting for DL-based methods, which are currently not widely used in the domain of phenotype prediction and might require new approaches to show their full potential. Hence, easyPheno supports a faster development of new prediction models, lowers the risk of errors and decreases testing efforts. Beyond that, easyPheno enables users to quickly benchmark newly developed prediction models against multiple comparison partners in a comparable and fair setup. Finally, also end users can benefit from this easy-to-extend design, making further prediction approaches available.

### 2.3 Systematic model assessment using synthetic data

To enable the evaluation of prediction models under pre-defined genotype-phenotype relationships, easyPheno allows the creation of synthetic phenotypes based on real genotypic data. Due to the pre-defined settings, this synthetic data is especially beneficial to evaluate the strengths and limitations of newly implemented prediction models. Supplementary Material contains an example on how to create synthetic data and analyze the results.

### 2.4 Further use cases

Furthermore, easyPheno provides functions to analyze prediction results, such as generating figures that provide a comparison of multiple predictors and phenotypes in a heat map. The framework supports the application of previously optimized models on new data, for instance to get predictions employing new samples of the same genotype-phenotype combination. Beyond that, easyPheno enables the integration of packages from R, thus allowing the enhancement of existing R code with new functionalities provided by our framework.

## 3 Implementation and features

As outlined, easyPheno’s easy-to-use and easy-to-extend design provides various benefits for end users and developers. Subsequently, we provide more details on easyPheno’s main features and their implementation.

### 3.1 Data pre-processing and management

easyPheno accepts various file formats for genotypic and phenotypic data, e.g. PLINK and CSV files. Details on the required format and accepted file types can be found in our Data Guide: http://bioweb.me/ep-dataguide. First, the data is transformed and saved in a unified format to enable consistent handling. In addition, common pre-processing techniques such as minor allele frequency filtering and three parameterizable types of data splits (train-validation-test, cross-validation with a separate test set and nested cross-validation) are implemented. Further, different genotype encodings are available through the preprocess module. All indices for data splits and pre-processing filters are stored in a dedicated HDF5 file referring to the usually large genotype data. This saves disk space and ensures a reproducible data preparation, even across different machines, and thus leads to comparable optimization results.

### 3.2 Prediction models and extendability

We distinguish between approaches that require a hyperparameter search (*_base_model*) as well as those with fixed parameters (*_param_free_base_model*). For both cases, we created a base class that contains useful methods for all related prediction models. Further, abstract methods and attributes are defined that have to be implemented in every child class. We provide classes already realizing most of the mandatory methods, e.g. the training loops, for the commonly used ML frameworks scikit-learn (*_sklearn_model*), PyTorch (*_torch_model*) and TensorFlow (*_tensorflow_model*). To implement a phenotype prediction model based on one of these widely used ML libraries, a user only needs to define two attributes, namely the default and possible nucleotide encodings, as well as two methods: the prediction model itself and potential hyperparameters including ranges. Hence, our design enables a straightforward and quick integration of further prediction models. In our online documentation, we show detailed information on how to integrate a new prediction model, including a step-by-step example in a video tutorial. Besides common genomic selection approaches, i.e. ridge regression best linear unbiased prediction (RR-BLUP) (Meuwissen *et al.*, 2001) and models from the Bayesian alphabet (Bayes A, Bayes B and Bayes C) (Habier *et al.*, 2011; Meuwissen *et al.*, 2001), our framework already includes various ML methods. These range from regularized linear respective logistic regression approaches (L1-, L2- and Elastic Net-regularization) over Support Vector Machine and Regression to the ensemble learners Random Forest and XGBoost. Additionally, we implemented three DL-based architectures, i.e. Multilayer Perceptron, Convolutional Neural Network (CNN) and Local CNN. We further demonstrate the integration of R packages by utilizing BGLR’s (Pérez and de los Campos, 2014) efficient implementation for Bayesian alphabet models (*_bayesfromR*). Detailed descriptions of all integrated prediction models can be found in our online documentation: http://bioweb.me/ep-models.

### 3.3 Automated optimization pipeline

We employ an automatic hyperparameter search using state-of-the-art Bayesian optimization via the Python package Optuna (Akiba *et al.*, 2019). Here, hyperparameters are suggested based on the performance of previous parameter sets. This might lead to a more efficient hyperparameter optimization compared to commonly used techniques, such as grid search or random search. Furthermore, for efficiency reasons, we stop the evaluation of non-promising parameter combinations based on intermediate cross-validation results. Beyond that, we use a consistent interface for prediction models with fixed parameters (*_param_free_base_model*) that only need to be fitted to the training data. This allows the user to employ both model types in the same pipeline.

### 3.4 Result analyses

easyPheno provides several methods to analyze prediction results, for which we also provide step-by-step tutorials in our documentation. For instance, we offer code to summarize the results of multiple optimization runs in a single CSV file. We further enable the generation of heat maps that contain prediction performances for multiple models and phenotypes.

### 3.5 Synthetic phenotype data

Using a linear mixed model, easyPheno offers to simulate phenotypic data based on real genotypic data. This simulation contains various parameterization options, for instance to model different numbers of causative markers and phenotypic distributions. More details regarding the underlying model and simulation options can be found in our online documentation: http://bioweb.me/ep-simulation. Hence, this enables the evaluation of a prediction model under pre-defined settings. To analyze how well the most influential markers are captured, easyPheno offers to plot the known effect sizes of simulated data against feature importances of several prediction models.

## 4 Documentation

We provide a comprehensive documentation for easyPheno at https://easypheno.readthedocs.io/. In addition to a complete API reference (http://bioweb.me/ep-api), we provide an Installation Guide for setting up easyPheno with Docker and as a Python package: http://bioweb.me/ep-install. Besides the tutorials mentioned above on how to integrate a new prediction model and analyze results, we included additional hands-on guides and videos at http://bioweb.me/ep-tutorials. For instance, we show how to use easyPheno in combination with Docker. Furthermore, with a code walkthrough video, we support users to comprehend easyPheno’s structure. All video tutorials guiding through the framework are embedded in our documentation but can also be found on YouTube: http://bioweb.me/ep-youtube.

## 5 Conclusion

With easyPheno, we present a comprehensive Python framework enabling the rigorous training, comparison and analysis of phenotype predictions for various models, ranging from common genomic selection approaches and classical ML to modern DL techniques. The framework allows state-of-the-art phenotype prediction also for non-programming-experts and fully automates the data pre-processing, the model optimization and the analysis of results. Further, the easy-to-extend design provides several benefits for bioinformaticians and invites other scientists to collaborate on easyPheno’s future. An additional advantage of our universal framework is the transfer to other prediction tasks with modest effort, as this only requires a re-implementation of the data pre-processing.

## Supplementary Material

vbad035_Supplementary_Data

## Data Availability

easyPheno’s source code is publicly available at: https://github.com/grimmlab/easyPheno. easyPheno is available as a Python package: https://pypi.org/project/easypheno/. A comprehensive documentation including various tutorials complemented with videos can be found at: https://easypheno.readthedocs.io/. In our GitHub repository, we provide exemplary data for testing easyPheno and debugging newly developed prediction models: http://bioweb.me/ep-testdata.
