# Supplementary material for: easyPheno: An easy-to-use and easy-to-extend Python framework for phenotype prediction using Bayesian optimization
Source: Bioinform Adv. 2023 Mar 22;3(1):vbad035. doi: 10.1093/bioadv/vbad035 (PMC10101695; doi:10.1093/bioadv/vbad035)
Supplement: vbad035_Supplementary_Data [file vbad035_supplementary_data.pdf]

Supplementary

easyPheno: A flexible and easy-to-use Python framework for phenotype prediction using Bayesian optimization

Florian Haselbeck [1,2,†], Maura John [1,2,†] and Dominik G. Grimm [1,2,3,*]

[1]Technical University of Munich, Campus Straubing for Biotechnology and Sustainability, Bioinformatics, 94315 Straubing, Germany, [2]Weihenstephan-Triesdorf University of Applied Sciences, Bioinformatics, 94315 Straubing, Germany, [3]Technical University of Munich, Department of Informatics, 85748 Garching, Germany

*To whom correspondence should be addressed.

†These authors have contributed equally to this work and share first authorship.

Exemplary workflow with real-world and simulated data

In this supplement, we will show how you can use easyPheno to perform state-of-the-art phenotype prediction. We will use both real data from *Arabidopsis thaliana* and synthetic phenotype data generated with our framework.

easyPheno provides two different workflows: It can either be installed as a Python package via pip and integrated into your pipeline, or it can be used as a command line tool inside a Docker container. In the following examples, we will focus on the Docker workflow and thus the easyPheno command line interface. For the pip workflow, we refer to the tutorial in our online documentation: http://bioweb.me/ep-tut-pip-package.

In the following, we assume that the repository has been successfully cloned, the Docker image has been built, and the dedicated container is running. We also assume that the container mounts a directory /myhome/ where our repository and the data are stored. See the easyPheno online documentation for a tutorial on creating a Docker image and setting up a Docker container: http://bioweb.me/ep-install-docker.

Our extensive online documentation provides various additional information that may be useful when using easyPheno, such as a full documentation of the API or several hands-on tutorials and video guides: https://easypheno.readthedocs.io/.

This supplementary focuses on how to use easyPheno. If you want to develop new phenotype prediction models, you may find our tutorial on integrating your own prediction model at http://bioweb.me/ep-tut-new-model as well as our code walkthrough video at http://bioweb.me/ep-code-walkthrough helpful.

1 Data

To run easyPheno, you need genotypic and corresponding phenotypic data. Our framework accepts different file types for both, which are explained in detail in the Data Guide of our online documentation, as well as the expected format: http://bioweb.me/ep-dataguide. The imputed genotype matrix should contain the IUPAC single-letter nucleotide code of your genotypic data, with each row representing an individual and each column representing a genetic marker. In addition, you will need sample and SNP identifiers. The phenotype matrix must contain the corresponding sample identifiers as the first column and the phenotype values as the remaining columns, with the trait name as the column header. Alternatively, easyPheno can create synthetic phenotypes based on the provided genotype matrix. For the following examples, we use genotypic and phenotypic data from *Arabidopsis thaliana* (Atwell *et al.*, 2010; Horton *et al.*, 2012). The original data can be downloaded from easyGWAS (Grimm *et al.*, 2017). To remove highly correlated markers, we applied linkage-disequilibrium pruning using PLINK v1.9 (Purcell *et al.*, 2007; Chang *et al.*, 2015) The resulting genotype matrix contains 1307 individuals and 43510 SNPs. Regarding the phenotypic data, we use the quantitative trait Flowering Time at 16 degrees (FT16) with 193 available samples. The preprocessed data are stored in our repository as atwell_ld_pruned.h5 and atwell_pheno_FT16.csv at https://github.com/grimmlab/easyPheno/tree/main/case_study.

The generation of the synthetic phenotype data, for which we use the same real-world genotype data as described above, is outlined below in section 3. For details on the underlying model and a step-by-step guide, we refer to our online documentation at http://bioweb.me/ep-simulation.

2 Workflow with real-world data

The following outlines a typical workflow for `easyPheno` with the real-world data described above.

2.1 Optimization pipeline

1. Navigate to the directory where the `easyPheno` repository is stored within the `Docker` container:

```
cd /myhome/easyPheno
```

2. To start the optimization run for the genotypic and phenotypic data described above, only a single-line command is required. We need to specify the data directory where our files are stored (`data_dir`), the name of our genotype (`genotype_matrix`) and phenotype file (`phenotype_matrix`) as well as the name of the phenotype (`phenotype`) within the phenotype matrix. We further set the directory in which we want to save our results (`save_dir`). In this example, we want to filter our genotypes for a minor allele frequency of 10% (`maf`). As a data split (`datasplit`), we want to use a cross-validation with an additional test set (`cv-test`). Finally, we need to specify all the models we want to optimize (`models`). In this tutorial, we choose Ridge Regression BLUP (`blup`), Bayes B (`bayesBfromR`), Support Vector Regression (`svm`), and a Multilayer Perceptron (`mlp`). Additionally, we set the number of trials for the Bayesian optimization to 100 (`n_trials`).

```
python3 -m easypheno.run --data_dir case_study/ --save_dir /myhome --genotype_matrix
↪  atwell_ld_pruned.h5 --phenotype_matrix atwell_pheno_FT16.csv --phenotype FT16 -maf 10
↪  --datasplit cv-test --models blup bayesBfromR svm mlp --n_trials 100
```

After executing this single-line command, the whole optimization pipeline is running. Some remarks regarding files and outputs that are generated:

- Once an optimization run starts, the following folders and files are created:
  - In the first run with a given genotype-phenotype combination, a dedicated index file is created and stored in the data directory (here: `atwell_ld_pruned-atwell_pheno_FT16-FT16.h5`). This file allows faster data loading in future runs and reproducible data splits across machines.
  - Dedicated result folders for each optimization run are created within the specified save directory. To distinguish similar runs, the folder name contains the real time (YYYY-MM-DD_hh-mm-ss) when the optimization started. In addition, we can find information on the optimization configuration, e.g., the datasplit and prediction models:
    `results/atwell_ld_pruned/atwell_pheno_FT16/FT16/`
    `cv-test_5-20_MAF10_bayesBfromR+svm+mlp+blup_YYYY-MM-DD_hh-mm-ss`
  - For each model optimized, a separate folder is created within the results directory containing several files that can be used for debugging and results analysis. This folder contains CSV files with the test results of the final model, a runtime overview, the unfitted model of the best trial and the corresponding validation results.
- More details, for example on all the different models implemented within `easyPheno`, can be found in the online documentation (`https://easypheno.readthedocs.io/`). An overview of all the different options you can set with `easyPheno's` optimization pipeline, is provided via the `help` command:

```
python3 -m easypheno.run --help
```

2.2 Result analysis

Once all models are optimized, we can analyze the results using the `postprocess` module of `easyPheno`

1. To create a results summary, we only need to specify the results directory at the level of the genotype matrix (`results_dir`) and the evaluation metric we want to visualize afterwards (`eval_metric`), in this case the explained variance:

```
python3 -m easypheno.postprocess.run_summarize_results --results_dir
↪  /myhome/results/atwell_ld_pruned/ --eval_metric explained_variance
```

   This creates an XLSX document (`results_summary_all_phenotypes_cv-test_5-20_MAF10.xlsx`) containing for each phenotype associated with the specified genotype and each optimized model the mean of common evaluation metrics as well as the process and real time. Additionally, a CSV file is created, only containing the specified metric (explained variance in this case). This file is needed to visualize the results in the next step.

2. To visualize the results in a heatmap, we need to specify the path to the CSV file created in the previous step (`results_summary_path`) and the directory where the plot should be saved (`save_dir`). The resulting plot, which also contains the results for the synthetic phenotypes that are based on the same genotype matrix (see Section 3) is shown in Figure 1. By default, `easypheno.postprocess.run_plot_results` generates a heatmap. However, there is an argument `plot` that can be used to call another plot function that you may want to implement.

```
python3 -m easypheno.postprocess.run_plot_results --results_summary_path
↪  /myhome/results/atwell_ld_pruned/Results_summary_all_phenotypes_cv-test_5-20_MAF10.csv
↪  --save_dir /myhome/results
```

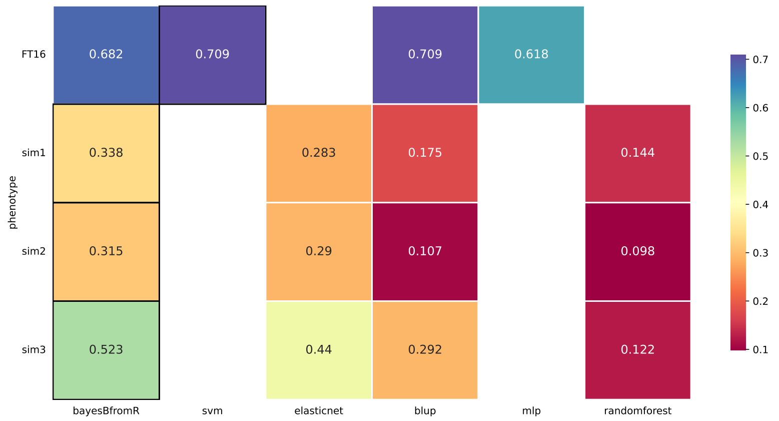

Fig. 1. Results of real-world and synthetic data shown in a heatmap: Each cell gives the explained variance ν that the prediction model given on the horizontal axis achieved for the phenotype specified on the vertical axis. The color of each cell ranging from dark red to dark blue represents the prediction performance. The best result for each phenotype is highlighted by a black frame around the cell. The blank cells indicate that the corresponding phenotype-model combination was not trained.

3 Workflow with synthetic data

The following describes a typical workflow with synthetic data.

3.1 Creation of synthetic phenotypes

First, we use `easyPheno's simulate` module to create synthetic phenotypes based on the genotype matrix described above. An overview of all different options when simulating phenotypes is provided via the `help` command:

```
python3 -m easypheno.simulate.run_synthetic_phenotypes --help
```

In this tutorial, we create three different synthetic phenotypes. For each, we need to specify the data directory where the genotype file is stored (`data_dir`) and the name of the genotype matrix to use (`genotype_matrix`). All simulations will be stored in a dedicated folder named after the genotype file (`atwell_ld_pruned/`) within the user-defined data directory.

1. For our first simulation, we want to filter the matrix for a minor allele frequency of 10% (`maf`). We further want to generate 500 samples (`number_of_samples`), and use an explained variance of 20% (`explained_variance`) and a heritability of 80% (`heritability`). By default, `easyPheno` uses one causal marker, 1000 SNPs to represent the polygenic background and simulates noise with a normal distribution:

```
python3 -m easypheno.simulate.run_synthetic_phenotypes --data_dir case_study/
↪  --genotype_matrix atwell_ld_pruned.h5 --number_of_samples 500 --explained_variance 20
↪  -maf 10 --heritability 80
```

2. For the second simulation, we choose again a maf of 10%, five causal markers (`number_causal_snps 5`) and a gamma-distributed noise (`distribution gamma`). By default, `easyPheno` simulates 1000 samples with a heritability of 70% and an explained variance of 30%.
3. As a third simulation, we use a maf of 10%, five causal markers, a heritability of 90% and default values for the remaining options.

The simulation files as well as the synthetic phenotypes within are named in the order they were created, e.g., the first file is called `Simulation_1.csv` and the phenotype within *sim1*. In addition to the phenotype files, an overview file is created which stores general information on all created synthetic traits. In a subfolder called `sim_configs`, the SNP identifiers of all used background and causal markers are stored together with their corresponding effect sizes. These files can be used to analyze how well certain models capture the most influential markers by comparing the feature importances of a model with the effect sizes. For more details on the resulting files, we refer to `easyPheno's` online documentation at `http://bioweb.me/ep-simulation`.

3.2 Optimization pipeline

The optimization pipeline is the same as for the real-world data shown in Section 2.1.

1. Before we start an optimization run, we need to copy the synthethic phenotype matrices into the same directory as the genotype matrix:

```
/myhome/easyPheno/case_study/atwell_ld_pruned$ cp Simulation_* ..
```

2. To start the optimization run for the simulated data, we again only need a single-line command similar to the real-world data. We need to specify the simulation file (`phenotype_matrix`) as well as the name of the synthetic trait (`phenotype`) within the simulation matrix. For the synthetic data, we choose to optimize Ridge Regression BLUP (`blup`), Bayes B (`bayesBfromR`), Elastic Net (`elasticnet`), and Random Forest (`randomforest`).

```
python3 -m easypheno.run --data_dir case_study/ --save_dir /myhome --genotype_matrix
↪  atwell_ld_pruned.h5 --phenotype_matrix Simulation_1.csv --phenotype sim1 -maf 10
↪  --datasplit cv-test --models blup bayesBfromR elasticnet randomforest --n_trials 100
```

To start the optimization for the other two simulations, we only need to change the name of the phenotype matrix and of the phenotype accordingly. All folders and result files that are created during an optimization run are similar to the ones for the real-world data (see Section 2.1).

3.3 Result analysis

The result analysis is similar to the experiments on real-world data, see Section 2.2. In addition, we are able to compare the effect sizes of the genetic markers and the feature importances assigned by certain prediction models, as we know the ground truth for the simulated data.

1. First, we can again generate a results summary as follows:

```
python3 -m easypheno.postprocess.run_summarize_results --results_dir
↪  /myhome/results/atwell_ld_pruned/ --eval_metric explained_variance
```

   This will generate the same files as explained in Section 2.2. As we use the same genotype matrix and data split for the real-world and synthetic phenotypes, the results will be summarized in the same files.
2. We further want to generate a heatmap summarizing the results for all optimized models and phenotypes. Again, as we use the same genotype matrix, data split and minor allele frequency filter, the results of the real-world and synthetic phenotypes are shown in the same plot shown in Figure 1

```
python3 -m easypheno.postprocess.run_plot_results --results_summary_path
↪  /myhome/results/atwell_ld_pruned/Results_summary_all_phenotypes_cv-test_5-20_MAF10.csv
↪  --save_dir /myhome/results
```

3. To analyze how well our optimized models capture the underlying architecture of the simulated phenotypes, we can visualize the feature importance of the models and the true effect sizes in scatter plots. We only need to specify the path to the results directory at the level of the genotype matrix (`results_dir`), the full path to the directory where the simulation configuration files are stored (`sim_config_dir`) and the save directory (`save_dir`). Note that not all models provide a feature importance.

```
python3 -m easypheno.simulate.run_results_analysis_synthetic_data --results_dir
↪  /myhome/results/atwell_ld_pruned --sim_config_dir case_study/atwell_ld_pruned/sim_configs
```

   This creates scatter plots for each model individually and one plot with all models combined. The combined scatter plot of the models we optimized is shown in Figure 2. Additionally, this function generates a CSV file with statistical information how well the influential markers are captured for each model (`Simulated_effect_sizes_vs_featimps_statistics_cv-test_5-20_MAF10.csv`).

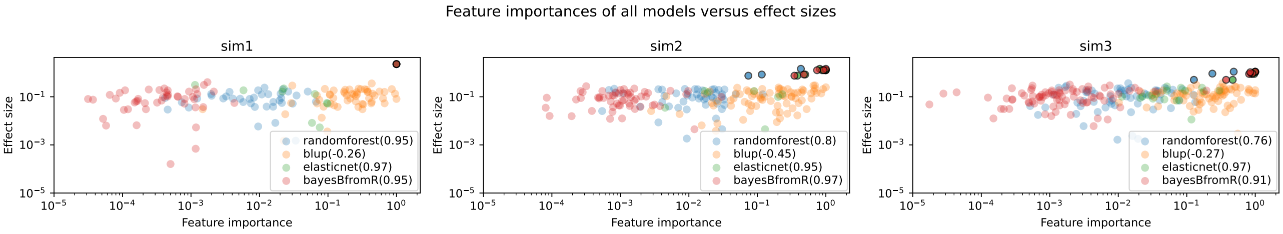

Fig. 2. Min-max normalized feature importances of Bayes B, Elastic Net, BLUP, and Random Forest in comparison with effect sizes on synthetic data: Each subplot shows the results of one of the three synthetic phenotypes on a logarithmic scale. All SNPs for which the effect size or the feature importance is zero are omitted. Causal markers are highlighted by a larger marker size and a black frame. The Pearson correlation coefficient of the effect sizes and feature importances is given in parenthesis for each model.

References

Atwell, S. *et al.* (2010). Genome-wide association study of 107 phenotypes in arabidopsis thaliana inbred lines. *Nature*, **465**(7298), 627–631.
Chang, C. C. *et al.* (2015). Second-generation plink: rising to the challenge of larger and richer datasets. *GigaScience*, **4**(1), s13742–015–0047–8.
Grimm, D. G. *et al.* (2017). easygwas: A cloud-based platform for comparing the results of genome-wide association studies. *The Plant cell*, **29**(1), 5–19.
Horton, M. W. *et al.* (2012). Genome-wide patterns of genetic variation in worldwide arabidopsis thaliana accessions from the regmap panel. *Nature genetics*, **44**(2), 212–216.
Purcell, S. *et al.* (2007). Plink: a tool set for whole-genome association and population-based linkage analyses. *The American journal of human genetics*, **81**(3), 559–575.
